# Comparison of State-of-the-Art Neural Network Survival Models with the Pooled Cohort Equations for Cardiovascular Disease Risk Prediction

**DOI:** 10.1186/s12874-022-01829-w

**Published:** 2023-01-24

**Authors:** Yu Deng, Lei Liu, Hongmei Jiang, Yifan Peng, Yishu Wei, Zhiyang Zhou, Yizhen Zhong, Yun Zhao, Xiaoyun Yang, Jingzhi Yu, Zhiyong Lu, Abel Kho, Hongyan Ning, Norrina B. Allen, John T. Wilkins, Kiang Liu, Donald M. Lloyd-Jones, Lihui Zhao

**Affiliations:** 1 Center for Health Information Partnerships, Northwestern University Feinberg School of Medicine, Chicago, IL USA; 2 Division of Biostatistics, Washington University in St. Louis, St. Louis, MO USA; 3 Department of Statistics and Data Science, Northwestern University, Chicago, IL USA; 4 Department of Population Health Sciences, Weill Cornell Medicine, New York, NY USA; 5 Department of Statistics, University of Manitoba, Winnipeg, MB Canada; 6 Department of Computer Science, University of California, Santa Barbara, CA USA; 7 Department of Preventive Medicine, Northwestern University Feinberg School of Medicine, Chicago, IL USA; 8 National Center for Biotechnology Information, National Library of Medicine, National Institute of Health, Bethesda, MD USA

**Keywords:** Artificial intelligence, Cardiovascular disease, Cox regression, Deep learning, Machine learning, Neural network, Pooled Cohort Equations, Predictive modeling, Survival analysis

## Abstract

**Background:**

The Pooled Cohort Equations (PCEs) are race- and sex-specific Cox proportional hazards (PH)-based models used for 10-year atherosclerotic cardiovascular disease (ASCVD) risk prediction with acceptable discrimination. In recent years, neural network models have gained increasing popularity with their success in image recognition and text classification. Various survival neural network models have been proposed by combining survival analysis and neural network architecture to take advantage of the strengths from both. However, the performance of these survival neural network models compared to each other and to PCEs in ASCVD prediction is unknown.

**Methods:**

In this study, we used 6 cohorts from the Lifetime Risk Pooling Project (with 5 cohorts as training/internal validation and one cohort as external validation) and compared the performance of the PCEs in 10-year ASCVD risk prediction with an all two-way interactions Cox PH model (Cox PH-TWI) and three state-of-the-art neural network survival models including Nnet-survival, Deepsurv, and Cox-nnet. For all the models, we used the same 7 covariates as used in the PCEs. We fitted each of the aforementioned models in white females, white males, black females, and black males, respectively. We evaluated models’ internal and external discrimination power and calibration.

**Results:**

The training/internal validation sample comprised 23216 individuals. The average age at baseline was 57.8 years old (SD = 9.6); 16% developed ASCVD during average follow-up of 10.50 (SD = 3.02) years. Based on 10 × 10 cross-validation, the method that had the highest C-statistics was Deepsurv (0.7371) for white males, Deepsurv and Cox PH-TWI (0.7972) for white females, PCE (0.6981) for black males, and Deepsurv (0.7886) for black females. In the external validation dataset, Deepsurv (0.7032), Cox-nnet (0.7282), PCE (0.6811), and Deepsurv (0.7316) had the highest C-statistics for white male, white female, black male, and black female population, respectively. Calibration plots showed that in 10 × 10 validation, all models had good calibration in all race and sex groups. In external validation, all models overestimated the risk for 10-year ASCVD.

**Conclusions:**

We demonstrated the use of the state-of-the-art neural network survival models in ASCVD risk prediction. Neural network survival models had similar if not superior discrimination and calibration compared to PCEs.

**Supplementary Information:**

The online version contains supplementary material available at 10.1186/s12874-022-01829-w.

## Background

Cox Proportional Hazards (Cox PH) model is widely used to quantify the effect of covariates in relation to time-to-event outcomes or to predict the survival time for a new individual [1]. Cox PH is a semi-parametric model, which consists of two main components: baseline hazard and multiplicative covariate effect in hazard ratio. The estimates of its regression coefficients are obtained through optimization of the partial likelihood function, which depends on both censored and uncensored individuals.

With the availability of large datasets and high-speed computational power, neural network algorithms have become increasingly popular. Neural networks have been successful when applied to unstructured data such as image recognition and text classification [2–7]. Compared to Cox PH, standard neural network architectures focus on predicting outcomes as a binary classification problem at a specific follow-up point. However, it is common in medical studies that individuals are lost to follow-up (censored data) before the failure or event time. Standard neural network models cannot train or test on these individuals. In 1995, Faraggi-Simon first combined neural network architectures with the Cox PH model to make use of censored information as well as to model non-linear features-outcome relations [8]. Since then, there has been increasing interest in incorporating neural network architectures in survival analysis. In current literature, there are two main ways of modeling time-to-event using neural networks: (i) adapting Cox PH model and using partial likelihood loss, e.g., Cox-nnet [9] and Deepsurv [10]; or (ii) discretizing survival time and using a heuristic loss function, e.g., Nnet-survival [11].

Atherosclerotic cardiovascular disease (ASCVD) is the leading cause of death globally [12]. Currently, some commonly used prediction models for ASCVD are based on Cox PH, such as the Framingham CHD risk score and its derivatives [13]. In recent years, the American College of Cardiology (ACC)/American Heart Association (AHA) guidelines developed new equations, i.e., the Pooled Cohort Equations (PCEs), to estimate 10-year ASCVD risk in non-Hispanic whites and African Americans [14]. The equations are developed based on datasets from several community-based epidemiology cohort studies. The PCEs are four race-, sex-specific and Cox PH based models. It is unclear whether neural network survival models can outperform PCEs for 10-year ASCVD risk prediction. In addition, it is unclear how different architectures of neural network survival models perform compared to each other. In this study, we compared the four race- and sex-specific PCEs with race- and sex-specific state-of-the-art neural network survival models: Nnet-survival, Deepsurv, and Cox-nnet [10, 11, 15] in primary ASCVD risk prediction. For fair comparison, we also included Cox PH models with all significant two-way interactions since this enables Cox PH to capture more complex relationships. For all models, we used the same seven predictors as in the PCEs. Our study is the first study to compare the state-of-the-art neural network survival models with PCEs in incident ASCVD prediction.

## Methods

### Model I, II: Pooled Cohort Equations, all two-way interaction Cox PH

PCEs are four Cox PH based models, each of which is for a specific race and sex group (white male, white female, black male, black female). Cox PH models the probability an individual experiences the event during a small-time interval given the individual is free of an event at the beginning of the time interval [1], which is also known as hazard rate. Specifically, the hazard function can be expressed as the follows:

1 $${\lambda }_{i}\left(t\right)={\lambda }_{0}\left(t\right)\mathrm{exp}\left({\beta }_{1}{X}_{i1}+\dots +{\beta }_{p}{X}_{ip}\right)={\lambda }_{0}\left(t\right)\mathrm{exp}({{\varvec{X}}}_{i}^{T}{\varvec{\beta}}),$$

where $t$ is the survival time, ${\lambda }_{0}\left(t\right)$ is the baseline hazard risk at time $t$, ${{\varvec{X}}}_{i}={\left[{X}_{i1},\dots ,{X}_{ip}\right]}^{T}$ contains the covariates for individual $i$, and ${\varvec{\beta}}={\left[{\beta }_{1},\dots ,{\beta }_{p}\right]}^{{\varvec{T}}}$ is the regression coefficient vector. The hazard function consists of two parts: baseline hazard ${\lambda }_{0}\left(t\right)$ and a hazard ratio or risk function $\mathrm{exp}\left({{\varvec{X}}}_{i}^{T}{\varvec{\beta}}\right)$. Cox PH assumes that the relative risk for each covariate (${\varvec{\beta}}$ in the equation) is constant over time. The estimate of ${\varvec{\beta}}$ is obtained by optimizing the Cox partial likelihood function as defined below:

2 $$l\left(\beta \right)={\sum }_{i:{\Delta }_{i}=1}\left({{\varvec{X}}}_{i}^{T}{\varvec{\beta}}-log{\sum }_{j:{Y}_{j}\ge {Y}_{i}}\mathrm{exp}\left({{\varvec{X}}}_{j}^{T}{\varvec{\beta}}\right) \right),$$

where ${\Delta }_{i}$ is the indicator for the occurrence of event and ${Y}_{j}$ is follow-up time for individual $j$.

In the PCEs, seven predictors were selected based on demonstrated statistical utility using prespecified criteria [14]. These predictors include age at baseline, systolic blood pressure (SBP), diabetes medical history, treatment for hypertension, current smoker, high density cholesterol and total cholesterol. The interactions between age at baseline and the other predictors were tested based on *p*-values. Only interactions that had significant *p*-values (< 0.05) were kept in the model. The PCEs demonstrated acceptable performance in derivation samples, with C-statistics for 10-year risk prediction of 0.80 in white females, 0.76 in white males, 0.81 in black females, and 0.70 in black males in 10 × 10 cross-validation [14].

To capture more complex relationships between predictors and ASCVD outcome, in the Cox PH-TWI model, we included all the two-way interactions of the 7 predictors in the model for each race and sex. We then retained only the interaction terms that had significant *p*-values for each race and sex.

### Models III and IV: Deepsurv and Cox-nnet

**Fig. 1:**
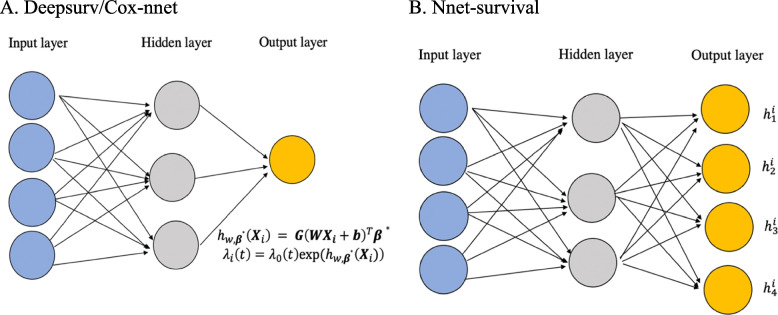
Frameworks for neural network survival models. The frameworks of Deepsurv/Cox-nnet and Nnet-survival are shown in A, B respectively. In Fig. 1A, the Deepsurv and Cox-nnet model output ${h}_{w}\left({{\varvec{X}}}_{i}\right)$ which is used to replace the log risk ${{\varvec{X}}}_{i}^{T}{\varvec{\beta}}$ in the Cox model. In Fig. 1B, the output layers generate ${h}_{j}^{i}$ which is the hazard for individual i at time j

Deepsurv and Cox-nnet are both adaptations of the standard Cox PH [10]. Instead of assuming the linear relationship between covariates and log-hazard, the Deepsurv and Cox-nnet models can automatically learn the non-linear relationship between risk factors and an individual’s risk of failure by its linear (i.e., multi-layer perceptron) and non-linear (activation functions) transformation. Specifically, the log-risk function ${{\varvec{X}}}_{i}^{T}{\varvec{\beta}}$ in the Cox equation as shown in Eq. (1) is replaced by the output from neural network ${h}_{w,{{\varvec{\beta}}}^{\boldsymbol{*}}}\left({{\varvec{X}}}_{i}\right)$, where ${{\varvec{\beta}}}^{\boldsymbol{*}}$ is the weight for the last hidden layer and $w$ is the weight for other hidden layers for neural network (see Fig. 1A):

$${h}_{w,{{\varvec{\beta}}}^{\boldsymbol{*}}}\left({{\varvec{X}}}_{i}\right) = {\varvec{G}}{\left({\varvec{W}}{{\varvec{X}}}_{j}+{\varvec{b}}\right)}^{T}{{\varvec{\beta}}}^{\boldsymbol{*}}.$$

The neural network optimizes the log-partial likelihood function similar to the standard Cox model by tuning parameters ${\varvec{W}},{{\varvec{\beta}}}^{\boldsymbol{*}}$:

$$l\left({\varvec{W}},{{\varvec{\beta}}}^{\boldsymbol{*}}\boldsymbol{ }\right)={\sum }_{i:{\Delta }_{i}=1}\left({h}_{w,{\varvec{\beta}}\boldsymbol{*}}\left({{\varvec{X}}}_{i}\right)-log{\sum }_{j:{Y}_{j}\ge {Y}_{i}}(\mathrm{exp}\left({h}_{w,{{\varvec{\beta}}}^{\boldsymbol{*}}}\left({{\varvec{X}}}_{j}\right)\right)\right).$$

Cox-nnet was proposed to deal with high dimensional features especially in genomic studies. To avoid overfitting, Cox-nnet introduces a ridge regularization term and subsequently, the partial log likelihood in Eq. (2) is extended as the following:

$$l\left( {\varvec{W}},{{\varvec{\beta}}}^{\mathbf{*}}\right)={\sum }_{i:{\Delta }_{i}=1}\left({\varvec{G}}{\left({\varvec{W}}{{\varvec{X}}}_{i}+{\varvec{b}}\right)}^{T}{{\varvec{\beta}}}^{\mathbf{*}}-log{\sum }_{j:{Y}_{j}\ge {Y}_{i}}\mathrm{exp}\left({\varvec{G}}{\left({\varvec{W}}{{\varvec{X}}}_{j}+{\varvec{b}}\right)}^{T}{{\varvec{\beta}}}^{\mathbf{*}}\right) \right)+\lambda \left(\parallel {{\varvec{\beta}}}^{\mathbf{*}}{\parallel }_{2}+\parallel {\varvec{W}}{\parallel }_{2}\right),$$

In addition to L_2_-regularizer, Cox-nnet also allows drop-out for regularization to avoid overfitting. The model is based on Theano framework, therefore, Cox-nnet can be run on a Graphics Processing Unit or multiple threads.

The Deepsurv model also allows the above-mentioned regularization techniques to avoid overfitting. In addition to that, Deepsurv adapted modern techniques to improve the training of the network such as introducing scaled Exponential Linear Units (SELU) as the activation function [8].

Although both the Cox-nnet and Deepsurv can learn the non-linear relationship between risk factors and the event risk, it is important to note that proportional hazard assumption still stands in the sense that the hazard ratio between any individual $\mathrm{i}$ and $j$ is constant over time.

### Model V: Nnet-survival

Nnet-survival is a fully parametric survival model that discretizes survival time. Nnet-survival is proposed to improve two main aspects of the neural network model that are adapted from Cox model: computational speed and the violation of the proportional hazard assumption. Neural network survival models that adapt from Cox model (e.g., Deepsurv, Cox-nnet) use partial likelihood function as the loss function to optimize. The partial likelihood function is calculated based on not only the current individual but also all the individuals that are at risk at the time point. This makes it difficult to use stochastic gradient descent or mini-batch gradient descent, both of which use a small subset of the whole dataset. Therefore, both Deepsurv and Cox-nnet may have slow convergence and cannot be applied to large datasets that run out of memory [9]. Nnet-survival was proposed to discretize time, which transforms the model into a fully parametric model and avoids the use of partial likelihood as the loss function. In Nnet-survival models, follow-up time is discretized to $n$ intervals. Hazard ${h}_{j}$ is defined as the conditional probability of surviving time interval $j$ given the individual is alive at the beginning of interval $j$. Survival probability at the end of interval $j$ can be then calculated as the following:

$${S}_{j}={\prod }_{i=1}^{j}\left(1-{h}_{i}\right).$$

The loss function is defined as the following:

$$L={h}_{j}{\prod }_{i=1}^{j-1}\left(1-{h}_{\left(i\right)}\right),$$

for individuals who fail at interval $j$, and

$$L={\prod }_{i=1}^{j-1}\left(1-{h}_{\left(i\right)}\right),$$

for individuals who are censored at the second half of interval $j-1$ or the first half of interval $j$.

There are two main architectures of Nnet-survival: a flexible version and a proportional hazards version. In the flexible version, output layers have $n$ neurons, where $n$ is the number of intervals and each output neuron represents the survival probability at the specific time interval given an individual is alive at the beginning of the time interval. In the proportional hazard version, the final layer only has a single neuron representing ${{\varvec{X}}}_{i}^{T}{\varvec{\beta}}$:

$${h}_{\beta }\left({{\varvec{X}}}_{{\varvec{i}}}\right)={{\varvec{X}}}_{i}^{T}{\varvec{\beta}},$$

In our study, the flexible version is used, with its architecture of the flexible version shown in Fig. 1B.

### Statistical analysis

In this study, we used the harmonized, individual-level data from 6 cohorts in the Lifetime Risk Pooling Project, including Atherosclerosis Risk in Communities (ARIC) study, Cardiovascular Health Study (CHS), Framingham Offsprinig study, Coronary Artery Risk Development in Young Adults (CARDIA) study, the Framingham Original study, and the Multi-Ethnic Study of Atherosclerosis (MESA). The first 5 cohort data were used for model development and internal validation, and the MESA data was used for external validation. We included individuals that meet the following criteria: (i) age between 40 to 79; and (ii) free of a previous history of myocardial infarction, stroke, congestive heart failure, or atrial fibrillation. ASCVD was defined as nonfatal myocardial infarction or coronary heart disease death, or fatal or nonfatal stroke (see [14] for details of selection criteria). All study individuals were free of ASCVD at the beginning of the study and were followed up until the first ASCVD event, loss to follow up, or death, whichever came first. We fit PCE, Cox PH with all two-way interactions (Cox PH-TWI), Nnet-survival, Deepsurv, and Cox-nnet models in white male, white female, black male, and black female participants. For comparison purposes, for all the models, we included the same predictors as used in the PCEs: age at baseline, systolic blood pressure (SBP), diabetes medical history, treatment for hypertension, current smoker, high density cholesterol (HDL-C) and total cholesterol. The details of the study start and end dates, study settings, and how the covariates were collected can be found in [14]. Individuals who had missing data at baseline were excluded from the study.

To obtain high performance neural network survival models, we manually tuned various hyper-parameters including learning rate, number of layers, number of neurons, number of epochs, batch size, momentum, optimizer, learning rate decay, batch normalization, L_2_ regularization, and dropout. More specifically, to tune the hyperparameters in the development dataset (the five training/internal validation cohorts), we split the data into 10 folds. We used nine folds of the data for training and the rest one-fold for evaluation. We used grid search to search through a range of hyperparameters and select the hyperparameter combination that generates the highest C-index in the one-fold evaluation dataset. After selecting the optimal hyper-parameters, we evaluated model performance through internal validation with 10 × 10 cross validation and external validation with the MESA data. To perform 10 × 10 cross-validation, we randomly partitioned the pooled cohort data into 10 equal-sized subsamples. Of the 10 subsamples, 9 subsamples were used as training data and the remaining single subsample was retained as the validation data for testing the model. Each of the subsamples is used in turn as the validation data. We repeated this process 10 times, during which 100 models were built. The average C-statistics and calibration plot of the 100 models were used as the final 10 × 10 cross-validation result. For PCE and Cox PH-TWI models, we refit the models in each of the cross-validated training samples for the internal 10 × 10 cross-validation to avoid overfitting. In each refit, we kept the original structure of the original PCE models and only updated the coefficients of the models. In the calibration plots, the observed and predicted events were shown in deciles [11]. For the external validation, we trained the model in the whole harmonized dataset (not including MESA cohort) and evaluated the model discrimination and calibration in the external MESA cohort. To compare whether the differences among C-statistics were significant in neural network models vs. PCE models, we performed significant test using method proposed by Uno et al. [16]. MESA is a more contemporary cohort that had lower CVD event rate compared to the earlier cohorts [14]. This difference could cause models to have poor calibration in MESA. To overcome this, we performed recalibration on all models using the method proposed by Pennells et al. [17]. Briefly, we first calculated rescaling factors that were needed to bring predicted risks in line with observed risks using regression model in MESA dataset. We then applied the rescaling factors to the original predicted risk and got recalibrated risk estimates for all participants.

Nnet-survival, Deepsurv, and Cox-nnet were implemented in python, version 3.7.3. Cox PH model was conducted using the “survival” package in R, version 3.6.0. C-statistics and the significant test in C-statistics between two competing risk prediction models were calculated using the “survC1” package in R, version 3.6.0 [16]. We chose 0.05 as the statistical significance level. Regression model for recalibration was performed using “scikit-learn” module in python, version 3.7.3 [18].

All data were de-identified, and all study protocols and procedures were approved by the Institutional Review Board at Northwestern University with a waiver for informed consent. All methods were performed in accordance with the relevant guidelines and regulations.

## Results

Overall, out of 26406 participants, 3190 (13.7%) had missing data at baseline. After excluding participants with missing data, there were 23216 participants left in total, including 8644 white male, 1354 black male, 10719 white female, 2499 black female individuals. The average age at baseline was 57.8 years old (SD = 9.6). Among these individuals, 16.0% developed ASCVD with average follow-up of 10.50 (SD = 3.02) years. The mean SBP value was 127.1 mmHg (SD = 21.0), the mean HDL-C value was 51.6 mg/dL (SD = 16.4), total cholesterol was 217.8 mg/dL (SD = 43.0). For binary predictors, 4.6% individuals had a history of diabetes, 26.0% individuals were current smokers, 31.6% individuals had treatment for hypertension. SBP, HDL-C, TOTCHL, history of diabetes, smoker percentage, age, and history of hypertension are all significantly different among the four race gender groups. More specifically, black males have the highest SBP, HDL-CL, percentage of diabetes history, percentage of smokers. The descriptive statistics for each race and sex group were shown in Table 1.

**Table 1:** Baseline characteristics for each race and sex group in training/internal validation dataset and external validation dataset

	**Overall**	**White male**	**Black male**	**White female**	**Black female**	***P*-value**
Training/internal validation dataset						
N	23216	8644	1354	10719	2499	
Number of Events, n (%)	3705 (16.0)	1788 (20.7)	300 (22.2)	1217 (11.4)	400 (16.0)	
Age (year), mean (SD)	57.8 (9.6)	58.0 (9.6)	57.3 (9.5)	58.0 (9.7)	56.4 (9.3)	< 0.001
SBP (mm Hg), mean (SD)	127.1 (21.0)	127.2 (19.5)	131.8 (21.5)	125.5 (21.6)	130.8 (22.7)	< 0.001
HDL-C (mg/dL), mean (SD)	51.6 (16.4)	43.8 (12.7)	49.7 (16.0)	56.8 (16.5)	57.5 (16.4)	< 0.001
TOTCHL (mg/dL), mean (SD)	217.8 (43.0)	212.1 (39.9)	208.6 (44.4)	223.9 (43.8)	216.0 (45.4)	< 0.001
HXDIAB, n (%)	1069 (4.6)	295 (3.4)	175 (12.9)	294 (2.7)	305 (12.2)	< 0.001
Smoker, n (%)	6035 (26.0)	2294 (26.5)	441 (32.6)	2723 (25.4)	577 (23.1)	< 0.001
RXHYP, n (%)	7326 (31.6)	2226 (25.8)	707 (52.2)	2951 (27.5)	1442 (57.7)	< 0.001
MESA external validation dataset						
N	4259	1194	799	1284	982	
Number of Events, n (%)	331 (7.8)	104 (8.7)	85 (10.6)	79 (6.2)	63 (6.4)	
Age (year), mean (SD)	61.6 (9.6)	61.9 (9.6)	61.5 (9.6)	61.5 (9.6)	61.3 (9.4)	0.446
SBP (mmHg), mean (SD)	126.3 (21.0)	123.7 (18.3)	130.0 (19.2)	121.8 (21.4)	132.4 (22.8)	< 0.001
HDL-C (mm/dL), mean (SD)	52.2 (15.5)	45.2 (12.1)	46.5 (12.5)	58.8 (15.8)	56.9 (15.6)	< 0.001
TOTCHL (mm/dL), mean (SD)	193.3 (35.7)	189.2 (34.4)	182.0 (34.6)	202.3 (34.4)	195.7 (36.5)	< 0.001
HXDIAB, n (%)	354 (8.3)	57 (4.8)	117 (14.6)	51 (4.0)	129 (13.1)	< 0.001
Smoker, n (%)	628 (14.7)	137 (11.5)	166 (20.8)	160 (12.5)	165 (16.8)	< 0.001
RXHYP, n (%)	1676 (39.4)	389 (32.6)	370 (46.3)	402 (31.3)	515 (52.4)	< 0.001

*Abbreviations*: *SD* standard deviation, *SBP* systolic blood pressure, *HDL-C* high density cholesterol, *TOTCHL* total cholesterol, *HXDIAB* history of diabetes, *RXHYP* history of hypertension

In the MESA external validation dataset, there were 4259 individuals in total. The average age at baseline was 61.6 years old (SD = 9.6). Among the 4259 individuals, 331 (7.77%) developed ASCVD with average follow-up years of 10.97 years old (SD = 2.48). Among these individuals, there were 1194 white males, 799 black males, 1284 white females, and 982 black females. All the baseline characteristics (among the 7 covariates) are significantly different among the four race gender groups, except for age. Similarly, we observed that black males have the highest SBP, HDL-C, percentage of diabetes history, percentage of smokers. Baseline characteristics of the study sample were shown in Table 1, stratified by race and sex group.

**Fig. 2:**
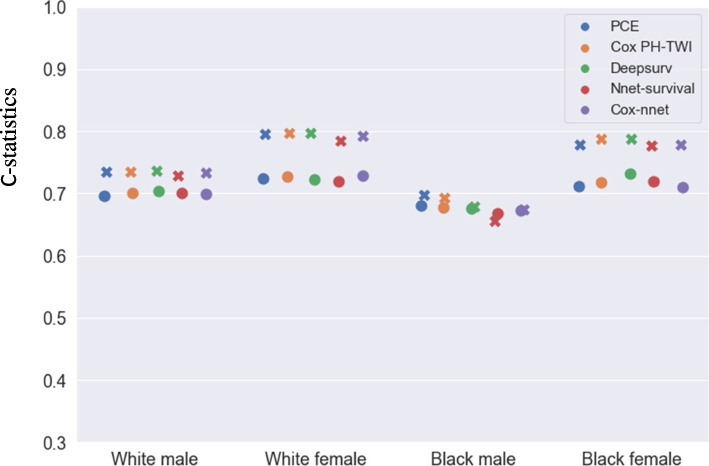
C-statistics for PCEs, Nnet-survival, Deepsurv, Cox-nnet, and Cox PH-TWI in 10 × 10 cross-validation and MESA external validation. The ‘x’ markers represent C-statistics in 10 × 10 cross-validation, the ‘o’ markers represent C-statistics in MESA external validation

In 10 × 10 cross validation, in the white male population (see Fig. 2 and supplemental Table 1), Deepsurv achieved the highest C-statistics (0.7371) among all the models. In the white female population, Deepsurv (0.7972) and Cox PH-TWI (0.7972) had the highest C-statistics. In the black male population, PCE had the highest C-statistics (0.6981). In the black female population, Deepsurv had the highest C-statistics (0.7886). The details of C-statistics for each model and race sex group were shown in Supplemental Table 1. In the external validation dataset, in white male population, Deepsurv had the highest C-statistics (0.7032). In white female population, Cox-nnet had the highest C-statistics (0.7282). In black male population, PCE had the highest C-statistics (0.6811). In black female population, Deepsurv (0.7316) had the highest C-statistics and this difference was statistically significant compared to PCE (*p* = 0.00, see Supplemental Table 1). Overall, when including results from both internal and external validations, Deepsurv had the highest C-statistics for five times followed by PCE which had the highest C-statistics for two times followed by Cox-nnet and Cox PH-TWI for one time. However, the difference between all neural network models vs. PCE are not significant except for Deepsurv in black females (see Supplemental Table 1).

**Fig. 3:**
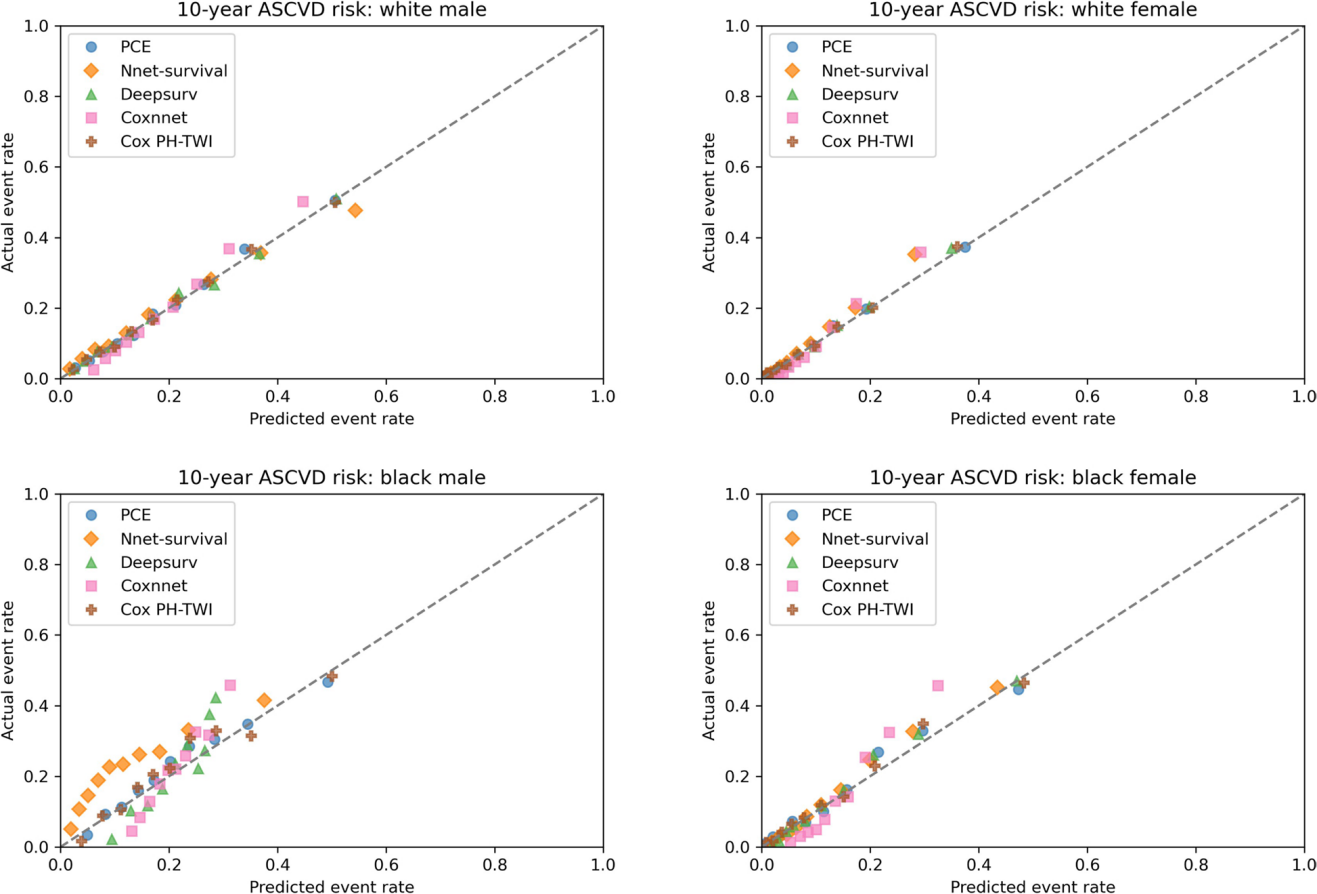
Kaplan–Meier Observed Event Rate and Predicted Event Rate for the ASCVD Outcome in the 10 × 10 cross-validation. For each model, we divided participants into 10 groups (decile) based on their sorted predicted event probability. Then, for each decile, mean observed event rate (Kaplan–Meier method) was plotted against mean predicted event rate. In a perfectly calibrated model, the predicted event rate would be the same as the observed event rate in each decile. This means that all points would be clustered around the blue identity line

**Fig. 4:**
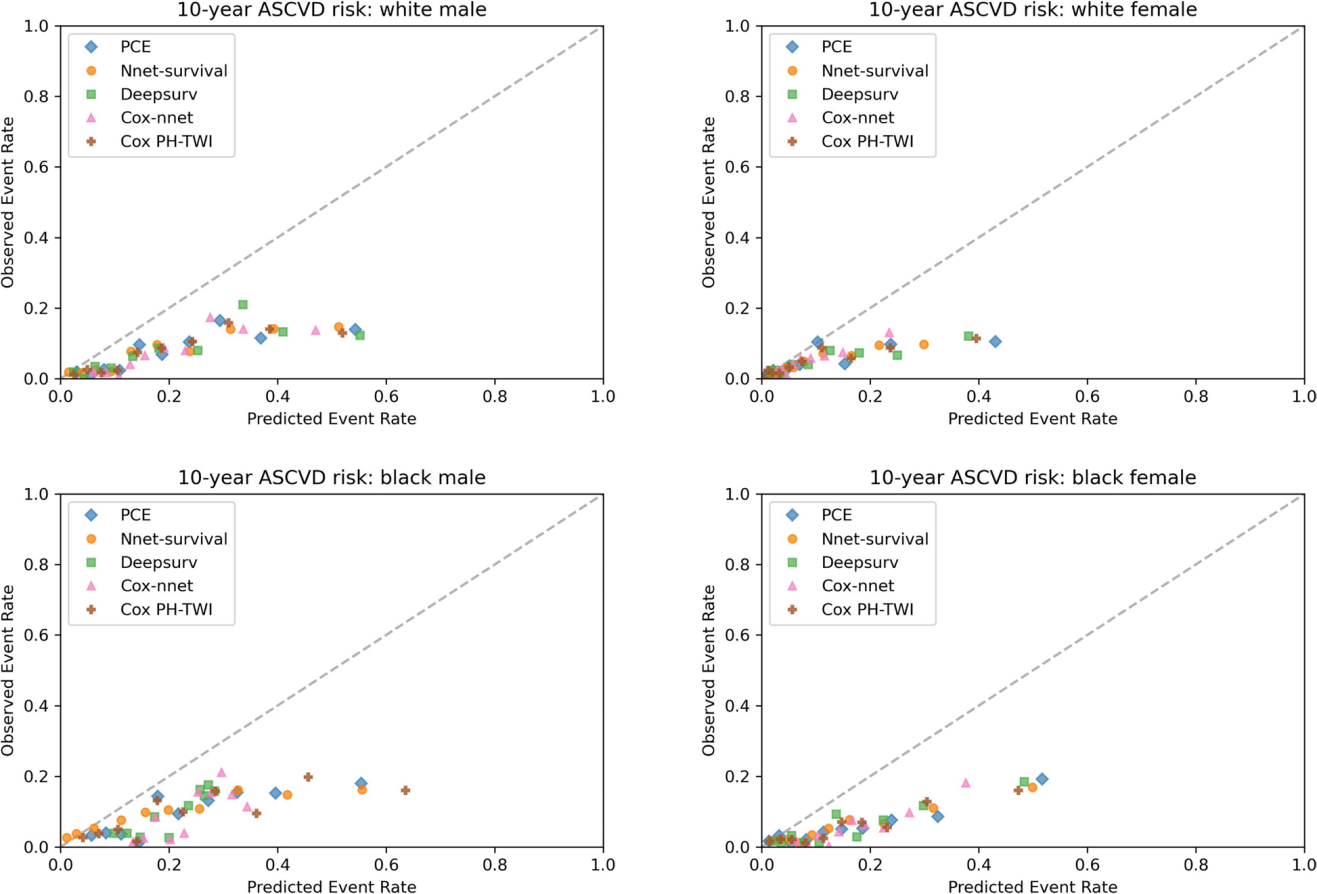
Kaplan–Meier Observed Event Rate and Predicted Event Rate for the ASCVD Outcome in the MESA Cohort. For each model, we divided participants into 10 groups (decile) based on their sorted predicted event rate. Then, for each decile, mean observed event rate (Kaplan–Meier method) was plotted against mean predicted event rate. In a perfectly calibrated model, the predicted event rate would be the same as the observed event rate in each decile. This means that all points would be clustered around the dotted identity line

**Fig. 5:**
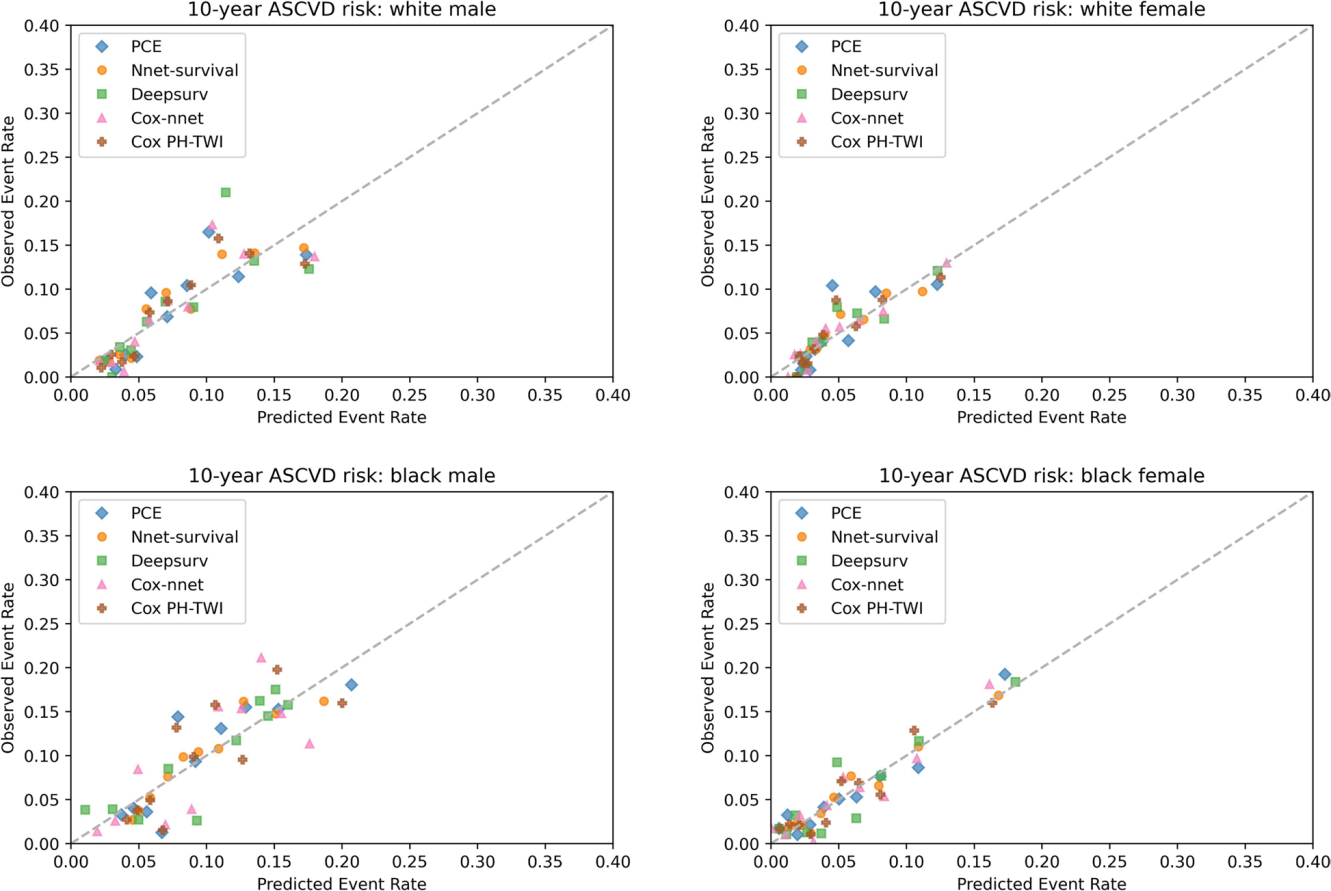
Kaplan–Meier Observed Event Rate and Recalibrated Predicted Event Rate for the ASCVD Outcome in the MESA Cohort. For each model, we divided participants into 10 groups (decile) based on their sorted recalibrated event rate estimates. Then, for each decile, mean observed event rate (Kaplan–Meier method) was plotted against mean recalibrated event rate estimates. In a perfectly calibrated model, the recalibrated event rate estimates would be the same as the observed event rate in each decile. This means that all points would be clustered around the dotted identity line

In terms of calibration, in 10 × 10 cross-validation (see Fig. 3), the calibration plot showed that all five models had similar calibration compared to PCE in white male and white female population. In black male population, PCE and Cox PH-TWI had better calibration compared to neural network models. In black female population, all models have better calibration than Cox-nnet. In the MESA external validation dataset, calibration plot showed that all five models overestimated the event rate among all race and sex groups. In white female and black female populations, all five models had similar overestimation with predicted event rate ranging from 0 to approximately 0.43 compared to 0 to approximately 0.18 in the observed event rate (see Fig. 4). In white male and black male populations, all five models had similar overestimation with predicted event rate ranging from 0 to approximately 0.6 compared to 0 to approximately 0.2 in the observed event rate (see Fig. 4). After recalibration by fitting linear regression models, over-estimations of event risk were greatly reduced in all models among all race and sex groups (see Fig. 5). The recalibration intercept and slope are summarized in Supplemental table 2.

## Discussion

In this study, we implemented state-of-the-art neural network survival models in predicting 10-year risk for a first ASCVD event. Our results showed that overall, when using the same predictors as in the PCEs, neural network survival models and PCE had comparable discrimination. Neural network survival models outperformed PCE in white male, white female, and black female population by slim margins. However, the difference is not statistically significant expect for Deepsurv in black female population. In terms of calibration, in the internal validation dataset, PCEs, Cox-PH TWI had good calibration across all race and sex populations, while the neural network models’ performance is not consistent. In external validation dataset, all models over-estimated the event rate in all four race-sex groups. Recalibration largely reduced the overestimation. Among different race gender groups, models in black males have relatively worse performance, which is consistent with the results from the PCE paper [1]. The number of African Americans, particularly men, is relatively low, which could potentially cause greater level of uncertainty with respect to the estimates. In the training dataset, the sample size for black males is 1354, which is much smaller than the white male (8644) and white female (8644) population. In addition, black male population has the highest ASCVD rate compared to other race gender groups. Our prior work showed racial differences in risks for first cardiovascular events and non-CVD death and competing risks analyses may yield somewhat different results than traditional Cox models and provide a complementary approach to examining risks for first CVD events [24].

Theoretically, among the different neural network survival models, Nnet-survival is faster in training than Deepsurv and Cox-nnet models. Nnet-survival’s loss function only relies on individuals in the current minibatch which allows mini-batch gradient descent while both Deepsurv and Cox-nnet require the entire dataset for each gradient descent update. On the other hand, the discretization of time-to-event in Nnet-survival leads to a less smooth predicted survival curve compared to Deepsurv and Cox-nnet.

In prior studies, Gensheimer et al. applied Cox PH, Nnet-survival, Deepsurv, and Cox-nnet in life expectancy prediction using the Study to Understand Prognoses and Preferences for Outcomes and Risks of Treatments (SUPPORT) dataset [11]. The dataset consisted of 9105 individuals and 39 predictors. The four neural network survival models generated similar C-statistics compared to the Cox PH model, which was consistent with our findings in ASCVD prediction. Both the SUPPORT dataset and our dataset had low dimension number of predictors. Several studies explored other machine learning methods for CVD prediction. Joo et al. [19] applied logistic regression, deep neural networks, random forests, and LightGBM to predict CVD as a binary outcome using the Korean National Health Insurance Service–National Health Sample Cohort dataset. The authors found that deep neural network had better performance (C-statistics = 0.7446) compared to the PCE (C-statistics = 0.7381) in that cohort. However, the ML models used more predictors (hemoglobin level, diastolic blood pressure, presence of proteinuria, serum aspartate aminotransferase, serum alanine aminotransferase, and total cholesterol) compared to the PCE. In another study, Dimopoulos et al. implemented KNN, random forest, and decision tree to predict CVD compared to the HellenicSCORE, a Cox regression based model [20]. Their results showed that ML models have comparable performance compared to the HellenicSCORE [21] using 5 and 13 same predictors respectively but were not able to outperform the baseline model.

Similar to other machine learning models, neural network models often show advantage in modeling non-linear complex relationships between predictors and outcome. We used the same predictors as the PCE, which are all well-studied risk factors of cardiovascular disease. The biologic basis for many of these variables has been well studied, and they are known to be independently and often linearly associated with risk of cardiovascular events. In this situation, simpler models might suffice since they can accurately capture a linear biologic relation without sacrificing interpretability. Similar conclusions were reached in data comparing three machine learning methods to a simpler logistic regression model for predicting death after acute myocardial infarction [22]. In the study, two of the 3 machine learning algorithms improved discrimination by a slim margin [22]. In the follow up editorial by Engelhard et al. [23], they mentioned that machine learning has been most impactful with complex data (e.g., high dimensional and difficult to summarize without substantial loss of information). There have been some explorations on using machine learning/deep learning with high dimensional features and/or longitudinal risk factors to improve CVD risk prediction. However, the findings are somewhat mixed in the literature. For instance, Zhao et al. implemented convolutional neural network and recurrent neural networks with long short-term memory using longitudinal electronic health records and genetic data and demonstrated significant improvement over the PCEs [24]. Dolezalova et al. trained both Cox PH and Deepsurv models using 608 variables derived from the UK Biobank. The two models achieved almost identical performance in C-index, although both models were superior to the Framingham risk score [25]. Taking together, with the same set of predictors as in the PCEs, our results show that the neural network survival models do not provide clinically meaningful improvement over the simpler and more interpretable PCEs. With more high dimensional complex data being readily accessible (e.g., repeated measurement data and imaging data in the electronic health records database), further research is needed to establish the clinical utility of neural network survival models and other machine learning/deep learning models for improving CVD risk prediction.

### Limitations

Our study has several limitations. First, the cohorts we used from the Lifetime Risk Pooling Project were the same cohorts used in the derivation of the PCEs. This may have led to some optimism in the performance of the PCEs. Second, the participants of our external validation cohort, MESA, were perhaps healthier than the general population. More importantly, they received intensive screening for subclinical CVD, which influenced health behaviors and preventive interventions including use of effective drug therapies; this may result in the lower event rate in MESA participants than what would have been predicted because of the use of effective preventive therapies selectively in higher-risk individuals.

## Conclusion

Neural network survival models can achieve comparable discrimination if not superior performance compared to the PCEs in 10-year time-to-ASCVD prediction in the white female, white male, black female, and black male population in our dataset. In future studies, high dimensional features and/or longitudinal risk factors should be considered to fully explore the benefits of neural network survival models for ASCVD risk prediction.

## Supplementary Information

**Additional file 1.**

## Data Availability

The Lifetime Risk Pooling Project data used in this study are not publicly available due to data use agreement with the National Heart, Lung, and Blood Institute (NHLBI). These data can be requested from the Biologic Specimen and Data Repositories Information Coordinating Center of NHLBI. Readers interested in the code used for this study may contact the corresponding author.

## Abbreviations

PCEs: Pooled Cohort Equations
ASCVD: Atherosclerotic cardiovascular disease
Cox PH: Cox Proportional Hazards
ACC: American College of Cardiology
AHA: American Heart Association
ARIC: Atherosclerosis Risk in Communities study
CHS: Cardiovascular Health Study
CARDIA: Coronary Artery Risk Development in Young Adults
SBP: Systolic blood pressure
