# Supplementary material for: Comparison of State-of-the-Art Neural Network Survival Models with the Pooled Cohort Equations for Cardiovascular Disease Risk Prediction
Source: BMC Med Res Methodol. 2023 Jan 24;23:22. doi: 10.1186/s12874-022-01829-w (PMC9872364; doi:10.1186/s12874-022-01829-w)
Supplement: Supplementary file 1 — Additional file 1. [file 12874_2022_1829_MOESM1_ESM.docx]

**Supplemental Table 1.** C-statistics for PCEs, Nnet-survival, Deepsurv, Cox-nnet, and Cox PH-TWI in 10x10 cross-validation and MESA external validation.

|  |  |  | **PCE** | **Cox PH-TWI** | **Deepsurv** | **Nnet-survival** | **Cox-nnet** |
| --- | --- | --- | --- | --- | --- | --- | --- |
| **White male** | 10x10 CV | C-statistic | 0.7349 | 0.7349 | **0.7371** | 0.728 | 0.733 |
|  | MESA | C-statistic  95% CI  P-value | 0.696  (0.653, 0.739)  - | 0.700  (0.652, 0.740)  0.31 | **0.703**  (0.661, 0.745)  0.16 | 0.701  (0.657, 0.745)  0.53 | 0.699  (0.656, 0.743)  0.59 |
| **White female** | 10x10 CV | C-statistic | 0.796 | **0.797** | **0.797** | 0.785 | 0.793 |
|  | MESA | C-statistic  95% CI  P-value | 0.724  (0.673, 0.774)  - | 0.728  (0.670, 0.778)  0.26 | 0.723  (0.666, 0.779)  0.67 | 0.719  (0.667, 0.771)  0.31 | **0.728**  (0.668, 0.788)  0.49 |
| **Black male** | 10x10 CV | C-statistic | **0.698** | 0.693 | 0.679 | 0.655 | 0.675 |
|  | MESA | C-statistic  95% CI  P-value | **0.681**  (0.625, 0.738)  - | 0.677  (0.630, 0.733)  0.66 | 0.676  (0.608, 0.744)  0.79 | 0.667  (0.609, 0.725)  0.43 | 0.673  (0.622, 0.724)  0.53 |
| **Black female** | 10x10 CV | C-statistic | 0.779 | 0.788 | **0.789** | 0.777 | 0.778 |
|  | MESA | C-statistic  95% CI  P-value | 0.711  (0.645, 0.777)  - | 0.718  (0.650, 0.772)  0.64 | **0.732**  (0.669, 0.794)  <0.01 | 0.719  (0.655, 0.782)  0.21 | 0.710  (0.649, 0.771)  0.91 |

C-statistics and 95% confidence intervals (CI) for all models and p-value for the difference of PCE models vs. other models. The highest C-statistics for each race and sex group are bolded. Abbreviations: PCE, Pooled Cohort Equation; Cox PH-TWI, all two way interaction Cox Proportional Hazard Model; CV, cross-validation; Cox PH, Cox Proportional Hazards model; MESA: Multi-Ethnic Study of Atherosclerosis.

**Supplemental Table 2.**  Model recalibration intercepts and coefficients for PCEs, Nnet-survival, Deepsurv, Cox-nnet, and Cox PH-TWI in MESA external validation.

| **Race-gender** | **Model** | **Intercept** | **Slope** |
| --- | --- | --- | --- |
| White male | PCE | 0.69 | 0.29 |
| White male | Nnet-survival | 0.68 | 0.30 |
| White male | Deepsurv | 0.70 | 0.28 |
| White male | Cox-nnet | 0.61 | 0.39 |
| White male | Cox PH-TWI | 0.68 | 0.30 |
| White female | PCE | 0.74 | 0.24 |
| White female | Nnet-survival | 0.66 | 0.33 |
| White female | Deepsurv | 0.58 | 0.41 |
| White female | Cox-nnet | 0.45 | 0.54 |
| White female | Cox PH-TWI | 0.71 | 0.27 |
| Black male | PCE | 0.64 | 0.34 |
| Black male | Nnet-survival | 0.34 | 0.26 |
| Black male | Deepsurv | 0.31 | 0.76 |
| Black male | Cox-nnet | 0.33 | 0.75 |
| Black male | Cox PH-TWI | 0.70 | 0.27 |
| Black female | PCE | 0.67 | 0.33 |
| Black female | Nnet-survival | 0.67 | 0.32 |
| Black female | Deepsurv | 0.61 | 0.39 |
| Black female | Cox-nnet | 0.52 | 0.51 |
| Black female | Cox PH-TWI | 0.66 | 0.34 |
